# Identifying Drivers Affecting the Spatial Distribution of Suitable Habitat for the Pine Wood Nematode (*Bursaphelenchus xylophilus*) in China: Insights From Ensemble Model and Geographical Detector

**DOI:** 10.1002/ece3.71433

**Published:** 2025-05-12

**Authors:** Liang Zhang, Jie Li, Chaokun Yang, Ping Wang, Guanglin Xie, Wenkai Wang

**Affiliations:** ^1^ MARA Key Laboratory of Sustainable Crop Production in the Middle Reaches of the Yangtze River (Co‐Construction by Ministry and Province), College of Agriculture Yangtze University Jingzhou China; ^2^ China National Cotton Research and Development Center, Xinjiang Academy of Agricultural Sciences Institute of Industrial Crops Urumqi China; ^3^ Institute of Entomology, College of Agriculture Yangtze University Jingzhou China

**Keywords:** *Bursaphelenchus xylophilus*, ensemble model, environmental drivers, optimal parameters‐based geographical detector (OPGD), spatial factors exploration

## Abstract

Biological invasions have become an important threat to global ecological security and forest health, and exploring the environmental driving mechanisms of invasive species is important for prevention and control. *Bursaphelenchus xylophilus* (Steiner and Buhrer, 1934), as a highly destructive invasive species, has its distribution and spread driven by a combination of various environmental factors. The study systematically evaluated the habitat suitability and key driving factors of *B. xylophilus* in the current period by applying an ensemble model and an optimized parameter‐based geographical detector. The results indicate that bioclimatic, vegetation indices, topographical features, and human activities are key environmental factors influencing the distribution of *B. xylophilus*, with highly suitable areas primarily located in southern, northern, and northeastern China. Meanwhile, the synergistic interaction between slope and population density (PD) significantly enhanced the suitability of *B. xylophilus* distribution, while the interaction between normalized difference vegetation index (NDVI) and global human influence index (GHII) exhibited a nonlinear weakening effect. Additionally, the habitat suitability of *B. xylophilus* increased with the expansion of isothermality, mean temperature of the wettest quarter, precipitation of the driest month, global human footprint, GHII, and PD, while it gradually decreased with the increase of UV‐B seasonality and NDVI. This study thoroughly explored the mechanisms by which various environmental factors influence the habitat suitability of *B. xylophilus*, revealing the complexity of regional driving factors. The findings not only provide theoretical support for predicting the ecological suitability of *B. xylophilus* but also offer scientific evidence for comprehensively analyzing the key factors affecting its distribution.

## Graphical abstract

While most studies focus on a single species distribution model, this study further analyzed the drivers of environmental factors by incorporating a geographic detector.

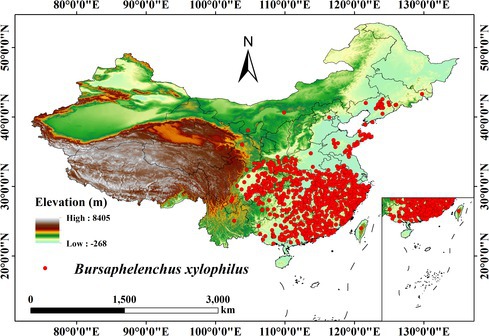

## 1 Introduction

Against the backdrop of global climate change and environmental degradation, the importance of forests has become increasingly prominent (Batavia and Nelson 2018). Forests, as the largest terrestrial ecosystems on Earth, play an irreplaceable role in maintaining the global ecological balance and absorbing and storing carbon emissions, while also supporting rich biodiversity by providing habitat and space for a wide variety of organisms to flourish (Kun et al. 2020; Jung et al. 2023). Pine trees, as an important silvicultural species, are widely planted around the world because of its adaptability, rapid growth, and diverse ecological services (Uprety et al. 2017). It not only provides timber production and other forest products for human consumption but also plays a key role in soil and water conservation, wind and sand control, and erosion reduction (Sharma et al. 2012). However, the health of pine trees is being seriously threatened by pine wilt disease (PWD), a devastating disease that has become a major problem globally affecting the health of pine trees and the stability of forest ecosystems, posing a serious challenge to the conservation and sustainable management of forest resources (Hu et al. 2021; Shi et al. 2022).

*Bursaphelenchus xylophilus* (Steiner and Buhrer 1934), the primary pathogen responsible for PWD, is considered one of the most severe threats facing global coniferous forest ecosystems (Tuomola et al. 2021). Native to North America, it spread to East Asia and Europe through international timber trade (Gruffudd et al. 2019). Currently, it is mainly distributed in Japan, North Korea, South Korea, the United States, Canada, Mexico, and Portugal (Zhang, Wang, et al. 2024a). Since its first introduction to China in 1982, *B. xylophilus* has rapidly spread across several provinces, including Jiangsu, Anhui, Shandong, Zhejiang, Guangdong, Hubei, Hunan, Hong Kong, Henan, Liaoning, Jiangxi, and Taiwan (Tang et al. 2021). According to the 2024 pest and disease area announcement by the National Forestry and Grassland Administration (NFGA), *B. xylophilus* has now spread to Jilin and Gansu, further intensifying the threat to China's forest ecosystems (Ouyang et al. 2022). *B. xylophilus* possesses a highly effective infectivity and lethality, and once it infects pine trees, it rapidly blocks the tree's vascular system, impairing water and nutrient flow, and leading to the rapid death of the tree (Chen et al. 2024). Additionally, according to statistics from 2017, the area of forest damage caused by *B. xylophilus* in China reached 86,000 ha, with economic losses amounting to approximately 19.5 billion RMB (Hu et al. 2021; Xiao et al. 2024). The invasion of *B. xylophilus* not only severely threatens forest resources but also has a profound impact on local ecological balance and biodiversity, and has become an important target of forest plant quarantine in China.

The ecological distribution and habitat suitability of *B. xylophilus* are driven by a combination of various environmental factors, including bioclimatic, topographic features, vegetation conditions, solar radiation, and human activity disturbances (Zhang, Wang, et al. 2024a). Among these, bioclimatic factors such as temperature and precipitation directly influence the survival and reproductive capacity of *B. xylophilus*, while topographic features such as slope and elevation significantly affect its spread rate and spatial distribution (Takai et al. 2001). Vegetation conditions and solar radiation affect the fitness of *B. xylophilus* by changing the structure of forest ecosystems, especially in areas with high forest cover, which can provide it with abundant host plant resources and further promote its reproduction and dispersal (Shi et al. 2022). Furthermore, human activities, particularly forestry, and urbanization, not only accelerate the spread of *B. xylophilus* but also modify its ecological adaptability, enabling it to survive and reproduce in a broader range of environments (Zhang, Wang, et al. 2024a). Therefore, accurate identification of potential high‐risk areas for *B. xylophilus* and in‐depth assessment of the driving role of key environmental factors can not only help to reduce the economic and ecological hazards caused by it, but also provide an important basis for ecological management and risk assessment in the relevant areas, so as to protect the health and stability of forest ecosystems.

In recent years, ecological niche models (ENMs) and geographical detectors have been widely applied in the ecological suitability analysis of invasive alien species, especially in assessing the potential distribution of species and analyzing environmental driver factors (Iannella et al. 2021; Zhang et al. 2022). ENMs combine environmental variables with known species occurrence data, utilizing machine learning algorithms to not only accurately predict the potential distribution of target species but also quantify the impact of environmental factors on species suitability (Feng et al. 2019; Gobeyn et al. 2019). The ensemble modeling (EM) approach, by combining multiple predictive models, is able to more accurately reflect the potential distribution range of species when faced with complex and heterogeneous environmental conditions, and this approach has been shown to have high predictive ability and accuracy in ecological research (Hao et al. 2019; Kaky et al. 2020). Geographical detectors, as a tool for quantifying the spatial explanatory power of environmental factors, can identify the key drivers of species distribution and their interactions, revealing how multiple factors jointly influence species ecological suitability (Wang et al. 2010; Song, Deng, et al. 2020). By decomposing spatial heterogeneity, GD can effectively clarify the complex relationships between environmental factors, providing a more comprehensive perspective for ecological predictions (Song, Wang, et al. 2020). However, most studies on the distribution of *B. xylophilus* mainly focused on a single modeling technique, and few studies have combined the ensemble model with the geographical detector to systematically analyze its suitability for distribution and driving mechanism, in order to comprehensively understand the appropriateness of its distribution and its driving mechanism (Ouyang et al. 2022; Xiao et al. 2024; Zhang, Wang, et al. 2024a). Therefore, exploring the potential of combining these two methods could provide a more accurate and comprehensive theoretical basis for predicting the distribution and assessing the ecological suitability of *B. xylophilus*.

This study aims to comprehensively assess the habitat suitability of *B. xylophilus* and its key environmental driving factors by combining ensemble modeling with optimal parameters geographical detector, providing a scientific basis for effective control of *B. xylophilus* spread. The objectives of this study are as follows: (1) to use ensemble models to identify high‐suitability areas for *B. xylophilus*, (2) to quantify the impact of different environmental factors on the distribution of *B. xylophilus*, and (3) to explore the influence of environmental factors and their interactions on the ecological suitability of *B. xylophilus* using the geographical detector. By employing ensemble modeling and integrating the optimized geographical detector, this study not only provides a more comprehensive understanding of the ecological suitability of *B. xylophilus* but also enables precise analysis of the mechanisms and interactions of key environmental driving factors. The results of this study will provide a theoretical basis for developing scientific control strategies for *B. xylophilus* and offer scientific support for regional ecological protection and policy formulation.

## 2 Materials and Methods

### 2.1 Collection of Species Occurrence Data

To generate the species distribution records required for the Biomod2 model, we collected distribution records of *B. xylophilus* from multiple reliable data sources, including: (1) Literature materials and online references (CNKI, https://www.cnki.net, accessed on August 20, 2024; WOS, https://www.webofscience.com/wos, accessed on August 25, 2024; and NACRC, http://museum.ioz.ac.cn, accessed on August 30, 2024); (2) National Forestry and Grassland Administration (NFGA) (http://www.forestry.gov.cn, accessed on August 2, 2024); (3) Global Biodiversity Information Facility (GBIF) (https://www.gbif.org/occurrence/search?taxon_key=8848401, accessed on November 29, 2024); (4) iNaturalist (https://www.inaturalist.org, accessed on November 29, 2024); (5) European and Mediterranean Plant Protection Organization (EPPO) Database (https://gd.eppo.int/taxon/BURSXY, accessed on November 20, 2024); (6) Center for Agriculture and Bioscience International (CABI) (https://www.cabidigitallibrary.org/doi/10.1079/cabicompendium.10448, accessed on November 25, 2024); and (7) Field survey data collected by researchers through GPS in various provinces and cities of China since 2013. Although several of these databases provide global occurrence data, we filtered and retained only those records located within the political boundaries of China to match the spatial focus of this study. After data cleaning and de‐duplication, a total of 2364 valid occurrence records were obtained.

**FIGURE 1:**
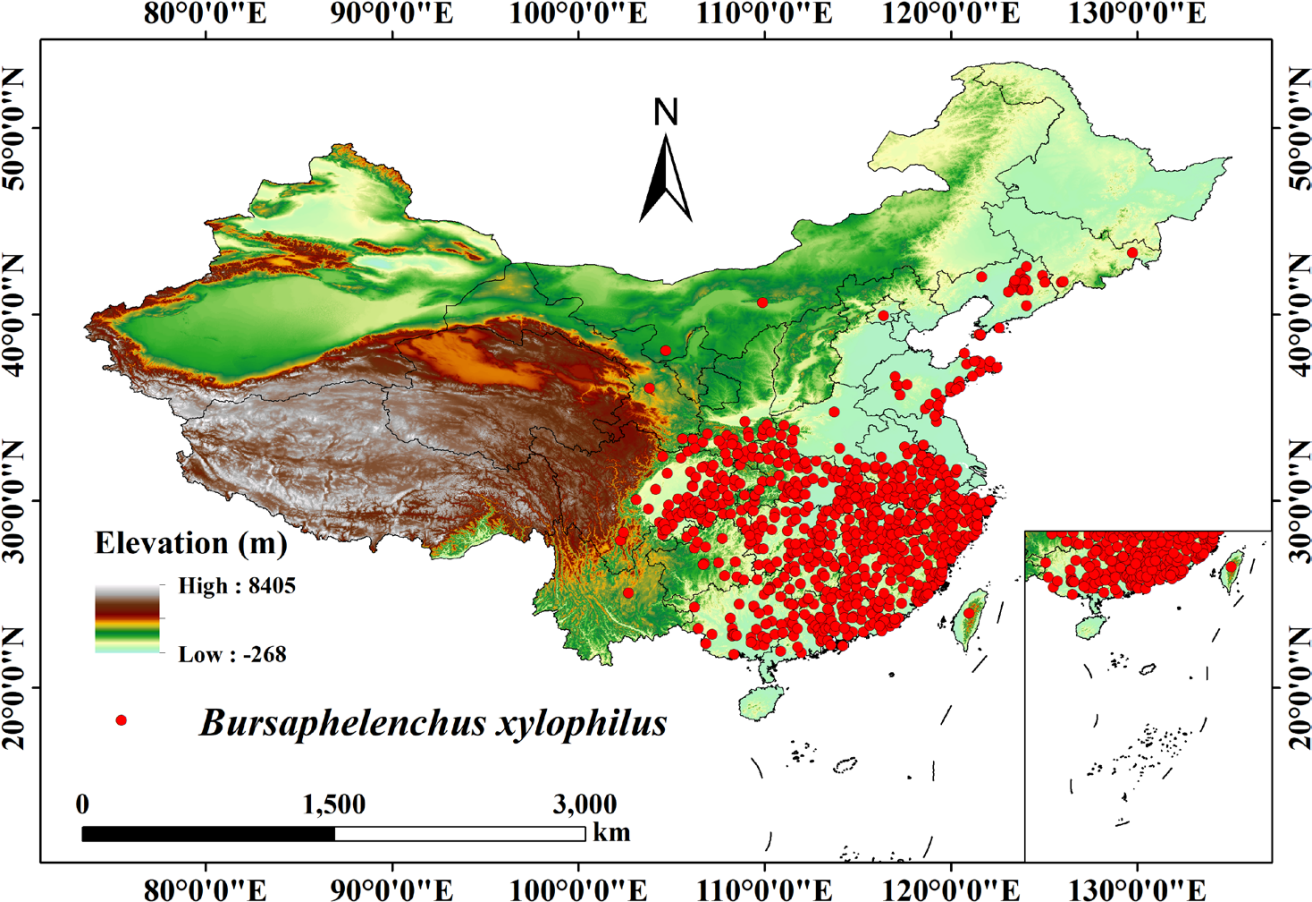
Occurrence records of *B. xylophilus*.

To avoid overfitting caused by spatial autocorrelation in the model, we used the “spThin” package (version 0.2.0) in R software (version 4.4.1) to sparsify thin the data, ensuring that only one distribution record was retained per grid cell (2.5 arc minutes). This effectively reduced the potential bias caused by spatial clustering, thereby improving the model's prediction accuracy and robustness (Aiello‐Lammens et al. 2015). Finally, we retained 767 distribution records for modeling (Figure 1).

### 2.2 Collection and Screening of Bioclimatic Variables

In this study, we downloaded 19 current bioclimatic variables with a resolution of 2.5 arc minutes from the WorldClim (version 2.1) climate database (https://www.worldclim.org, accessed on December 20, 2023). These variables encompass key climatic factors such as temperature, precipitation, and humidity, which are primary determinants of habitat suitability for species. We also downloaded elevation data for 2.5 arc minutes from the WorldClim database and extracted slope and aspect data using ArcGIS Map (version 10.8.1) software. Solar radiation data were obtained from the Helmholtz Centre for Environmental Research (https://www.ufz.de, accessed on February 5, 2024). The normalized difference vegetation index (NDVI) and enhanced vegetation index (EVI) data were downloaded from the Resource and Environmental Science Data Platform (https://www.resdc.cn, accessed on January 20, 2024). These datasets are based on MODIS 16‐day 250‐m‐resolution continuous time series data products, and the maximum value synthesis method was used to generate vegetation indices for 2023. Furthermore, we downloaded data related to human activities from the Socioeconomic Data and Applications Center (https://sedac.ciesin.columbia.edu, accessed on January 15, 2024), including the global human influence index (GHII), global human footprint (GHF), and population density (PD). Finally, we standardized all 33 environmental variables to a uniform format using the “Resample” and “Extract” tools in ArcGIS Map software for subsequent analysis and modeling (Wu et al. 2018).

**FIGURE 2:**
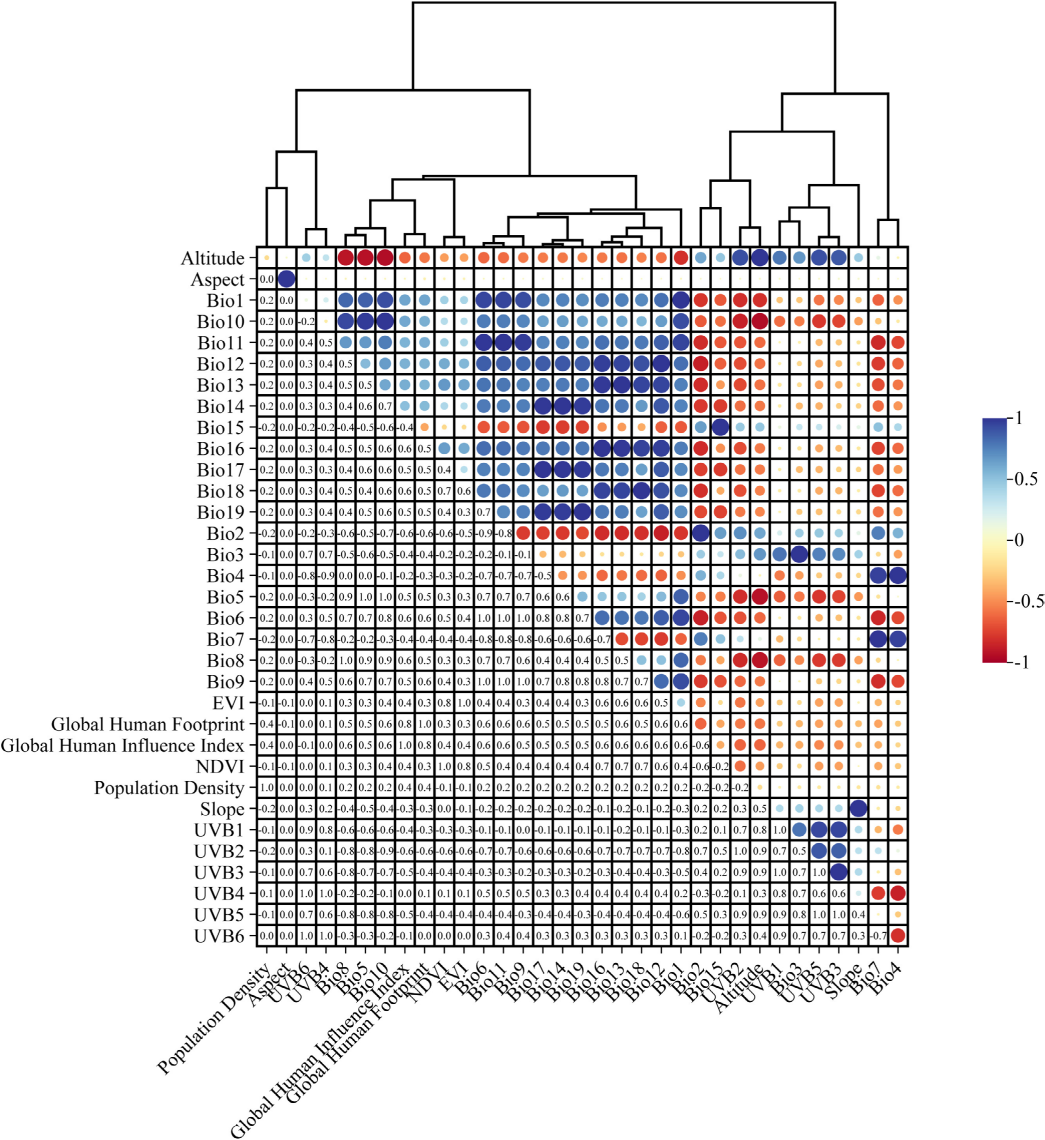
Pearson correlation coefficients among 33 environmental variables.

Variable selection is crucial in species distribution modeling, as high correlations between environmental factors can lead to autocorrelation and multicollinearity, thereby affecting the prediction accuracy and reliability of the model (Ayenewa et al. 2024). To address these potential issues, we performed Pearson correlation analysis on the 33 bioclimatic variables using the “car” (version 3.1.2) and “usdm” (version 2.1.7) packages in R software (Figure 2). Strong correlations between two or more environmental variables may significantly reduce model accuracy, and we subsequently used a stepwise approach to eliminate variables with a variance inflation factor (VIF) greater than 10. This approach effectively mitigated the issue of multicollinearity, ensuring that no excessive linear dependencies existed among the selected environmental variables, thus enhancing the stability and predictive capability of the model. Ultimately, 14 relatively independent environmental factors with significant influence on model prediction were selected (Table 1).

**TABLE 1:** Screening of 33 environmental variables.

Types	Abbreviation	Environmental variables	Operation (|*r*| > 0.8)
Bioclimate	Bio1	Annual mean temperature (°C)	Eliminate
	Bio2	Mean diurnal range (°C)	Eliminate
	Bio3	Isothermality	Retain
	Bio4	Temperature seasonality	Retain
	Bio5	Maximum temp of warmest month (°C)	Eliminate
	Bio6	Minimum temp of coldest month (°C)	Eliminate
	Bio7	Temperature annual range (°C)	Eliminate
	Bio8	Mean temp of wettest quarter (°C)	Retain
	Bio9	Mean temp of driest quarter (°C)	Eliminate
	Bio10	Mean temp of warmest quarter (°C)	Eliminate
	Bio11	Mean temp of coldest quarter (°C)	Eliminate
	Bio12	Annual precipitation (mm)	Eliminate
	Bio13	Precipitation of wettest month (mm)	Eliminate
	Bio14	Precipitation of driest month (mm)	Retain
	Bio15	Precipitation seasonality (mm)	Retain
	Bio16	Precipitation of wettest quarter (mm)	Eliminate
	Bio17	Precipitation of driest quarter (mm)	Eliminate
	Bio18	Precipitation of warmest quarter (mm)	Retain
	Bio19	Precipitation of coldest quarter (mm)	Eliminate
Topography	Altitude	Elevation (m)	Eliminate
	Aspect	Aspect	Retain
	Slope	Slope	Retain
Radiation	UVB1	Annual mean UV‐B	Eliminate
	UVB2	UV‐B seasonality	Retain
	UVB3	Mean UV‐B of highest month	Eliminate
	UVB4	Mean UV‐B of lowest month	Eliminate
	UVB5	Sum of UV‐B radiation of highest quarter	Eliminate
	UVB6	Sum of UV‐B radiation of lowest quarter	Eliminate
Vegetation	NDVI	Normalized difference vegetation index	Retain
	EVI	Enhanced vegetation index	Retain
Human activities	GHF	Global human footprint	Retain
	GHII	Global human influence index	Retain
	PD	Population density	Retain

### 2.3 Algorithms, Construction, and Validation of Ensemble Models

This study employs the “Biomod2” package (version 4.2.6–2) to construct ensemble models. The package provides a comprehensive framework for building and validating species distribution models, offering multiple algorithms for this purpose (Swets 1988; Hao et al. 2019). Using the ensemble modeling method, we combined the predictions of 12 different models through a weighted average (Wmean), which enhanced the predictive accuracy and robustness of the model. The individual models available in the “Biomod2” package include generalized linear models (GLM), generalized additive models (GAM), multivariate adaptive regression splines (MARS), flexible discriminant analysis (FDA), random forest (RF), maximum entropy model (MAXENT), generalized boosted models (GBM), classification tree analysis (CTA), artificial neural networks (ANN), surface range envelope (SRE), random forest with downsampling (RFD), and extreme gradient boosting (XGBOOST). Each of these algorithms has its own strengths and limitations when dealing with ecological data, and combining the outputs of multiple models can help to improve the robustness of the prediction results, which can adapt to the diverse characteristics of complex ecosystems.

In this study, we used AUC (area under the receiver operating characteristic curve) and TSS (true skill statistic) values as performance metrics to evaluate and validate the accuracy of the models. AUC is an important metric for measuring model accuracy and predictive performance, quantifying the model's classification ability and evaluating its overall performance. The AUC value ranges from 0 to 1, with values closer to 1 indicating better predictive performance, meaning the model distinguishes better between the species' actual distribution areas and nondistribution areas (Allouche et al. 2006). TSS is an index based on a combination of model sensitivity (quantification of omission error) and specificity (quantification of commission error) and provides a more comprehensive evaluation of the model's predictive capability (Liu et al. 2011). The TSS value ranges from −1 to 1, with values closer to 1 indicating better model performance, while values closer to 0 or negative indicate poor model performance. An AUC value close to 1.0 indicates the model's ability to distinguish suitable from unsuitable areas for the species, while a TSS value close to 1.0 further validates the model's robustness across different classification thresholds, reducing the likelihood of false classifications (Allouche et al. 2006; Poisot 2023). Therefore, by combining AUC and TSS, two complementary metrics, we can more comprehensively assess the model's reliability and predictive performance.

To ensure the reliability of the predictions, we first performed a preliminary analysis of these 12 models, selecting only a single model with AUC > 0.9 and TSS > 0.8 as the basis for the final ensemble model. In the modeling process, we used 80% of the species occurrence records as a training set and 20% as a test set, and 1000 pseudo‐absence points were randomly generated to balance the sample size. To improve the confidence of the models, 10 replications of each model were performed and cross‐validation methods (*k* = 10) were used to train the models to reduce each model's variance. Additionally, the automatic tuning strategy (tuned) provided by the Biomod2 package was used to optimize model parameters and further improve predictive performance. Finally, based on the ensemble model's output, we calculated the contribution of each environmental variable to the model and plotted the response curves of key variables on species survival probabilities, providing a comprehensive analysis of the impact of environmental variables on species distribution.

### 2.4 Predicting Habitat Suitability of *B. xylophilus* Using Ensemble Modeling

In this study, habitat suitability prediction layers for *B. xylophilus* were generated using the optimal ensemble model. The predictions were represented as a continuous TIFF raster, with the value of each raster pixel representing the probability (*p*) of *B. xylophilus* presence at that location, ranging from 0 to 1000, thus reflecting habitat conditions from least to most suitable (Zhang, Yang, et al. 2024). These predictive layers provide an intuitive and scientifically sound basis for assessing the suitable distribution of *B. xylophilus*, and can provide a more complete picture of habitat preferences and potential distribution patterns of *B. xylophilus*.

### 2.5 Optimal Parameter‐Based Geographical Detectors Model

Geographical detector is an effective method of spatial statistical analysis, widely used in a variety of fields such as ecology, geography, and public health, to identify and quantify the explanatory power of different drivers on spatial distribution patterns (Wang et al. 2010; Song, Deng, et al. 2020; Li et al. 2022). The method assesses explanatory power by comparing the variance of the dependent variable between different levels of categorical factors, thus revealing whether the spatial heterogeneity of the dependent variable is caused by a particular factor. It primarily consists of factor detectors, interaction detectors, ecological detectors, and risk detectors. This study utilized the optimal parameters geographical detector model (OPGD) to analyze the spatial explanatory relationship between environmental variables and the suitability of *B. xylophilus*.

#### 2.5.1 Single‐Factor Detector Analysis

Factor detector is used to assess the explanatory power of each environmental variable *X* on the target variable *Y* (i.e., the species suitability probability). Its core function is to measure the degree of influence of *X* on the spatial distribution of *Y* by calculating the *q*‐value. The q‐value ranges from 0 to 1 and represents the proportion of the total variance of *Y* that can be explained by the classification of *X*. Higher q‐values indicate greater explanatory power, implying that the pattern of the spatial distribution of the probability of appropriateness is highly dependent on the hierarchical structure of the environmental variables.

The calculation formula is as follows:

$$q=1-\frac{\sum_{h=1}^{L}N_{h}\cdot \sigma_{h}^{2}}{N\cdot \sigma^{2}}$$

Where: The q‐value ranges from 0 to 1, with a higher value indicating a stronger explanatory power of the environmental factor for habitat suitability. L refers to the number of categories in environmental factors. $N_{h}$ and $\sigma_{h}^{2}$ represent the sample size and variance of the h class, respectively.

In ecological niche modeling, especially in the context of invasive species risk assessment, identifying environmental variables with high q‐values is essential for understanding which climatic or ecological factors most significantly influence the species' potential distribution. This information provides crucial insights for environmental monitoring, habitat conservation, and the development of targeted management strategies.

To enhance the effectiveness of the factor detector, this study attempted multiple discretization methods for each continuous variable, including “equal”, “natural”, “quantile”, “geometric”, and “sd”, and combined with different numbers of classifications (3–7 classes) to generate multiple discretization schemes. The discretization scheme that produced the highest *q*‐value was ultimately chosen to capture the heterogeneity of spatial stratification, which in turn led to the identification of key variables with significant effects on the spatial distribution of species suitability.

#### 2.5.2 Interaction Detector Analysis

The interaction detector is used to evaluate the type and intensity of interactions between two environmental factors (Table 2). By analyzing the variations of q (X1), q (X2), and q (X1∩X2), the interaction types of the factors are determined, including “Independent,” “Enhance, nonlinear,” “Enhance, bi‐,” “Weaken, nonlinear,” and “Weaken, uni‐.” In this study, the method was used to analyze whether different environmental factors act together to enhance or weaken their effects on the probability of habitat suitability for *B. xylophilus*. This analysis not only helped to clarify the importance ranking of environmental factor interactions but also revealed complex ecological mechanisms that could not be detected by single‐factor analysis.

**TABLE 2:** Type of interaction detector result.

Interaction types	Basis of discrimination
Enhance, nonlinear	*q* (X_1_∩X_2_) > *q* (X_1_) + *q* (X_2_)
Independent	*q* (X_1_∩X_2_) = *q* (X_1_) + *q* (X_2_)
Enhance, bi—	*q* (X_1_∩X_2_) > Max [*q* (X_1_) + *q* (X_2_)]
Weaken, uni—	Max [*q* (X_1_), *q* (X_2_)] > *q* (X_1_∩X_2_) > Min [*q* (X_1_), *q* (X_2_)]
Weaken, nonlinear	*q* (X_1_∩X_2_) < Min [*q* (X_1_) + *q* (X_2_)]

#### 2.5.3 Ecological Detector Analysis

The ecological detector is used to assess whether there are significant differences in the distribution of species' habitat suitability across different categories of environmental variables. By comparing the mean habitat suitability within different subregions and conducting statistical significance tests, it can be verified whether different environmental factors significantly influence the spatial distribution of habitat suitability. The method works by performing pairwise comparisons of means between different strata (i.e., categories or intervals) of an environmental factor. It employs an *F*‐test (variance analysis) to evaluate the null hypothesis that the means of habitat suitability among different strata are equal. If the null hypothesis is rejected, it indicates that the environmental factor in question exerts a significant influence on the spatial heterogeneity of the species' habitat suitability. The results help identify not just which variables matter, but also where and to what extent they influence species distribution patterns, thereby offering critical guidance for ecological conservation, species management, and predictive modeling.

The calculation formula is as follows:

$$F=\frac{N_{X_{1}}\left(N_{X_{2}}-1\right)\mathrm{SS}W_{X_{1}}}{N_{X_{2}}\left(N_{X_{1}}-1\right)\mathrm{SS}W_{X_{2}}}$$

$$\mathrm{SS}W_{X_{1}}=\sum_{h=1}^{L1}N_{h}\delta_{h}^{2}$$

$$\mathrm{SS}W_{X_{2}}=\sum_{h=1}^{L_{2}}N_{h}\delta_{h}^{2}$$

where $\mathrm{SS}W_{X_{1}}$ and $\mathrm{SS}W_{X_{2}}$ are the sum of variance of strata formed by $X_{1}$ and $X_{2}$, $N_{X_{2}}$ denotes the sample size of these two factors, h, $L_{1}$, and $L_{2}$ represent the stratified number of the factors $X_{1}$ and $X_{2}$, respectively. Using the hypothesis $H_{0}$ that $\mathrm{SS}W_{X_{1}}$=$\;\mathrm{SS}W_{X_{2}}$, $H_{0}$ is rejected if the factors $X_{1}$ and $X_{2}$ are significantly different in influencing the spatial distribution of grasshoppers, and where *Y* represents significant difference and *N* represents no significant difference.

#### 2.5.4 Risk Detector Analysis

The risk detector is used to compare the average feature differences between subregions of different environmental factors, assessing whether there are significant spatial risk distribution differences. This analysis helps to identify high‐risk areas and further optimize conservation and management measures.

Through the above analysis, the main driving factors influencing the spatial distribution of *B. xylophilus* can be effectively identified. This allows for the exploration of interactions between environmental factors and the assessment of the distribution characteristics of high‐risk areas for the species, providing a scientific basis for the formulation of species management and conservation strategies.

The calculation formula is as follows:

$$t=\frac{Y_{h=1}-Y_{h=2}}{{\left[\frac{\mathrm{Var}\left(Y_{h=1}\right)}{n_{h}=1}+\frac{\mathrm{Var}\left(Y_{h=2}\right)}{n_{h}=2}\right]}^{1/2}}$$

where $Y_{h}$ denotes the mean value of Y in subregion h, $n_{h}$ is the number of samples in subregion h, and Var is variance.

## 3 Results

### 3.1 Model Evaluation

**FIGURE 3:**
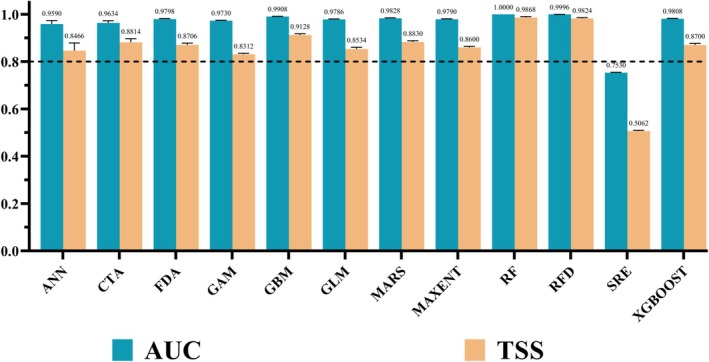
Evaluation of accuracy of 12 single models. Note: ANN (Artificial neural networks), CTA (Classification tree analysis), FDA (Flexible discriminant analysis), GAM (Generalized additive models), GBM (Generalized boosted models), GLM (Generalized linear models), MARS (Multivariate adaptive regression splines), MAXENT (Maximum entropy model), RF (Random forest), RFD (Random forest model with a down‐sampling method), SRE (Surface range envelope of profile models), and XGBOOST (Extreme gradient boosting).

This study evaluated the accuracy of the ensemble model in predicting the suitable distribution of *B. xylophilus* using AUC and TSS values. After running the model 10 times, the average AUC and TSS values of the 12 individual models were obtained. The results showed that the average AUC and TSS values for the SRE model were both below 0.8, failing to meet the minimum requirements for modeling. As a result, this model was excluded from the ensemble model. Ultimately, 11 individual models were selected to construct the ensemble model, including ANN, CTA, FDA, GAM, CBM, GLM, MARS, MAXENT, RF, RFD, and XGBOOST (Figure 3). The constructed ensemble model demonstrated high predictive performance, with an AUC value of 0.991 and a TSS value of 0.906 (Table 3). These results indicate that the model has high accuracy and reliability, and is capable of effectively predicting and validating the potential distribution of *B. xylophilus*.

**TABLE 3:** Model accuracy evaluation.

Sensitivity	Specificity	TSS	ROC
0.957	0.949	0.906	0.991

### 3.2 Contribution and Response Curves for Environmental Variables

**FIGURE 4:**
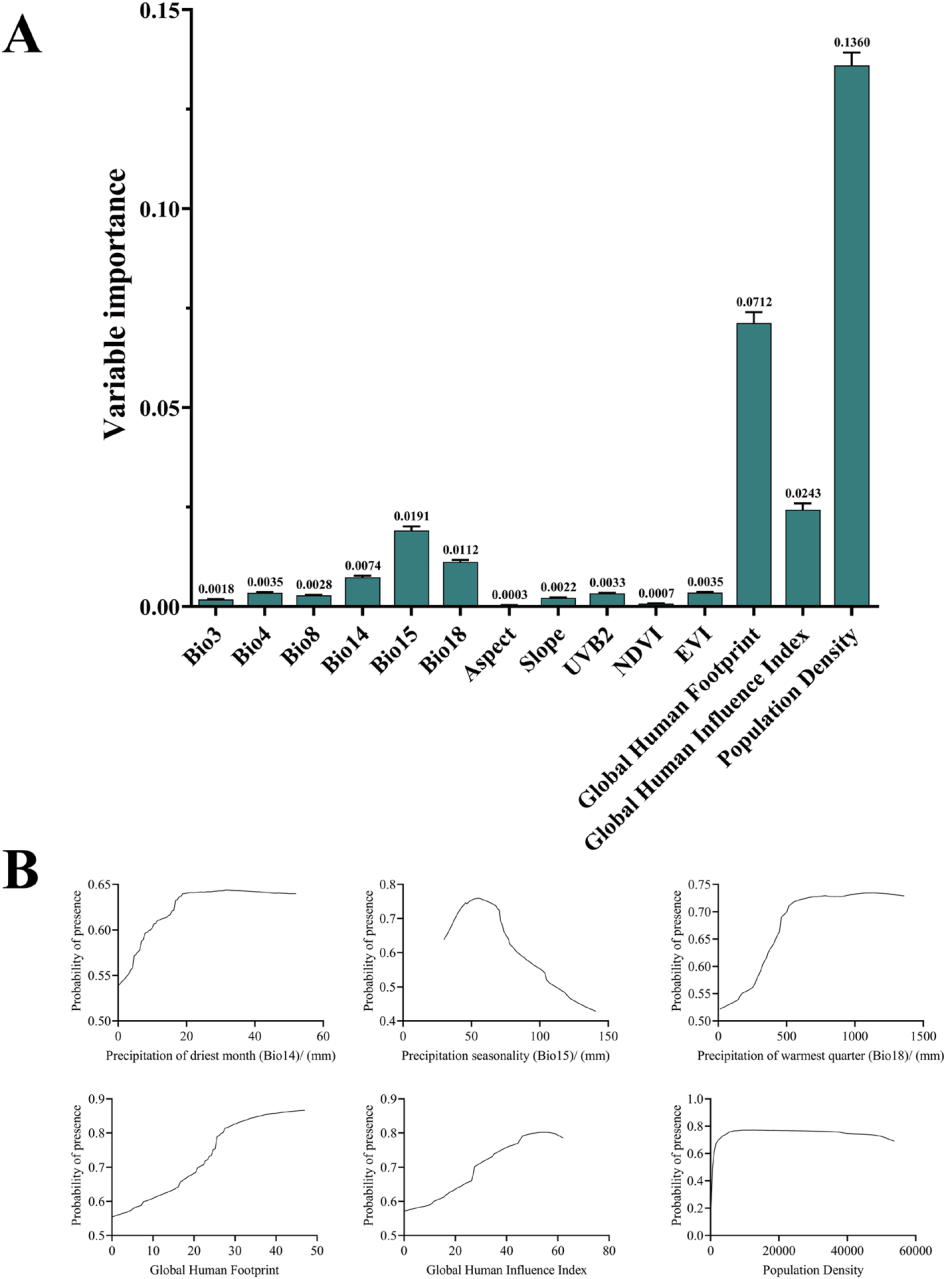
(A) Contribution of environment variables; (B) Response curve of the environment variable.

According to the importance evaluation of bioclimatic variables by the ensemble model, it was found that PD is the most significant environmental factor influencing the potential distribution of *B. xylophilus*, with an importance score of 0.1360, playing a decisive role in species distribution prediction. This was followed by GHF with an importance score of 0.0712, while Aspect had the lowest importance score of 0.0003 (Figure 4A). These results suggest that human activity, vegetation index, topographic features, and bioclimatic factors collectively affect the distribution pattern and suitability of *B. xylophilus*.

Furthermore, response curves further revealed the relationship between environmental factors and the habitat suitability of *B. xylophilus* (Figure 4B). The habitat suitability of *B. xylophilus* significantly increases with higher values of precipitation of driest month (Bio14), precipitation of warmest quarter (Bio18), GHF, and GHII. Whereas the response curve of precipitation seasonality (Bio15) indicates the existence of an optimal interval for the suitability of *B. xylophilus* for precipitation. When Bio15 reaches 54.57 mm, the habitat suitability is maximized (*p* = 0.76). However, when precipitation exceeds this threshold, excessively high rainfall may have adverse effects on the ecological adaptability of *B. xylophilus*, limiting its distribution range and habitat suitability.

### 3.3 Habitat Suitability for the Current Period

**FIGURE 5:**
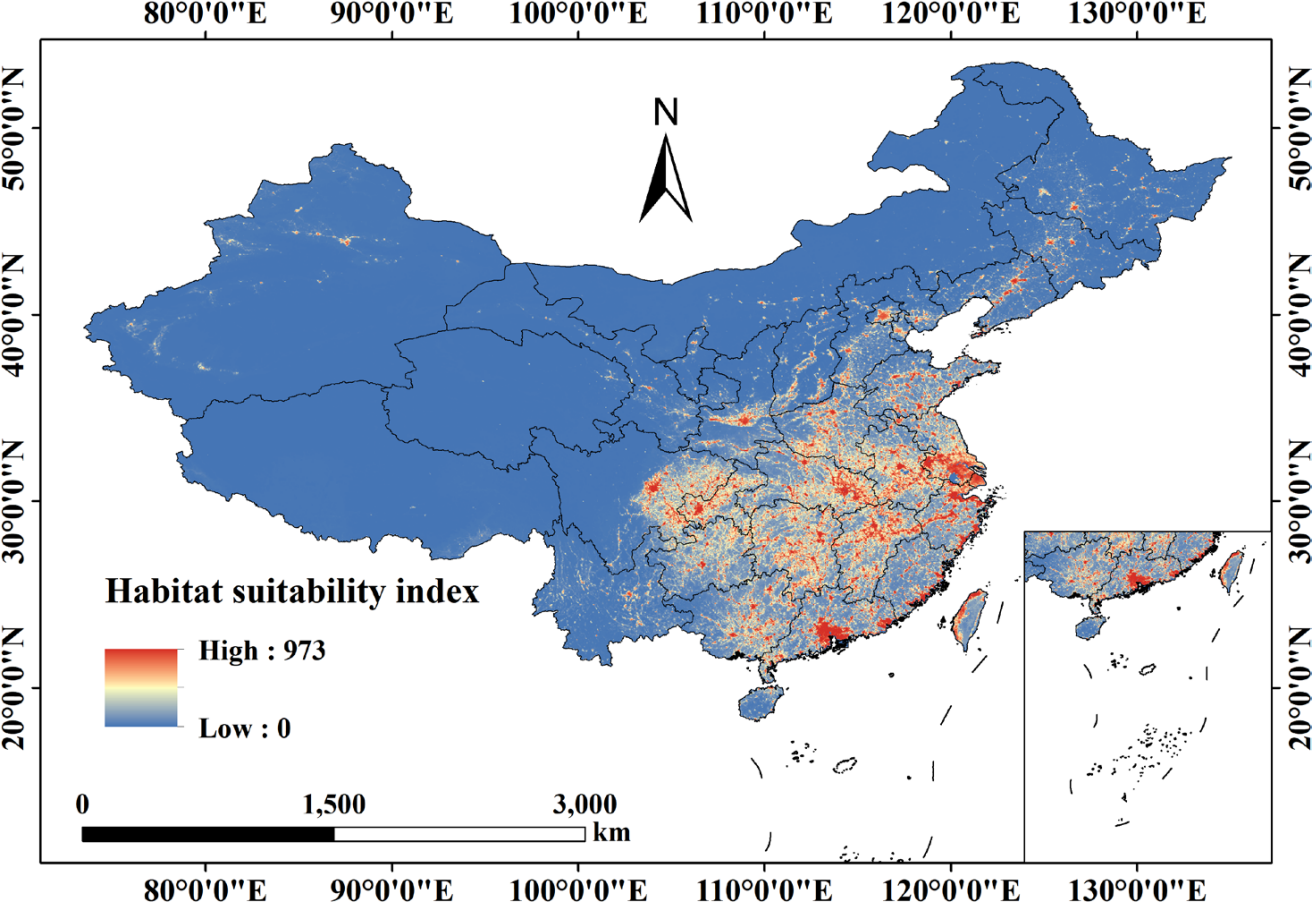
Habitat suitability of *B. xylophilus* in China under current environmental conditions.

The ensemble model was used to predict the current distribution suitability of *B. xylophilus* (Figure 5), with prediction probabilities ranging from 0 to 1000. The results show that areas with higher suitability for *B. xylophilus* are mainly concentrated in Northeast, North, and South China, including Beijing, Tianjin, Hebei, Shanxi, Liaoning, Jilin, Heilongjiang, Shanghai, Jiangsu, Zhejiang, Anhui, Fujian, Jiangxi, Shandong, Henan, Hubei, Hunan, Guangdong, Guangxi, Hainan Island, Chongqing, Sichuan, Guizhou, Yunnan, Shaanxi, Hong Kong, Macau, and Taiwan. Additionally, the predicted distribution area by the ensemble model aligns closely with the actual observed distribution, further validating the accuracy and practicality of the model. This indicates that the ensemble model we built can accurately reflect the potential distribution suitability of *B. xylophilus*.

### 3.4 Analysis of Factors Driving the Variation of Suitability of *B. xylophilus*

#### 3.4.1 Discretization of Continuous Variables

**FIGURE 6:**
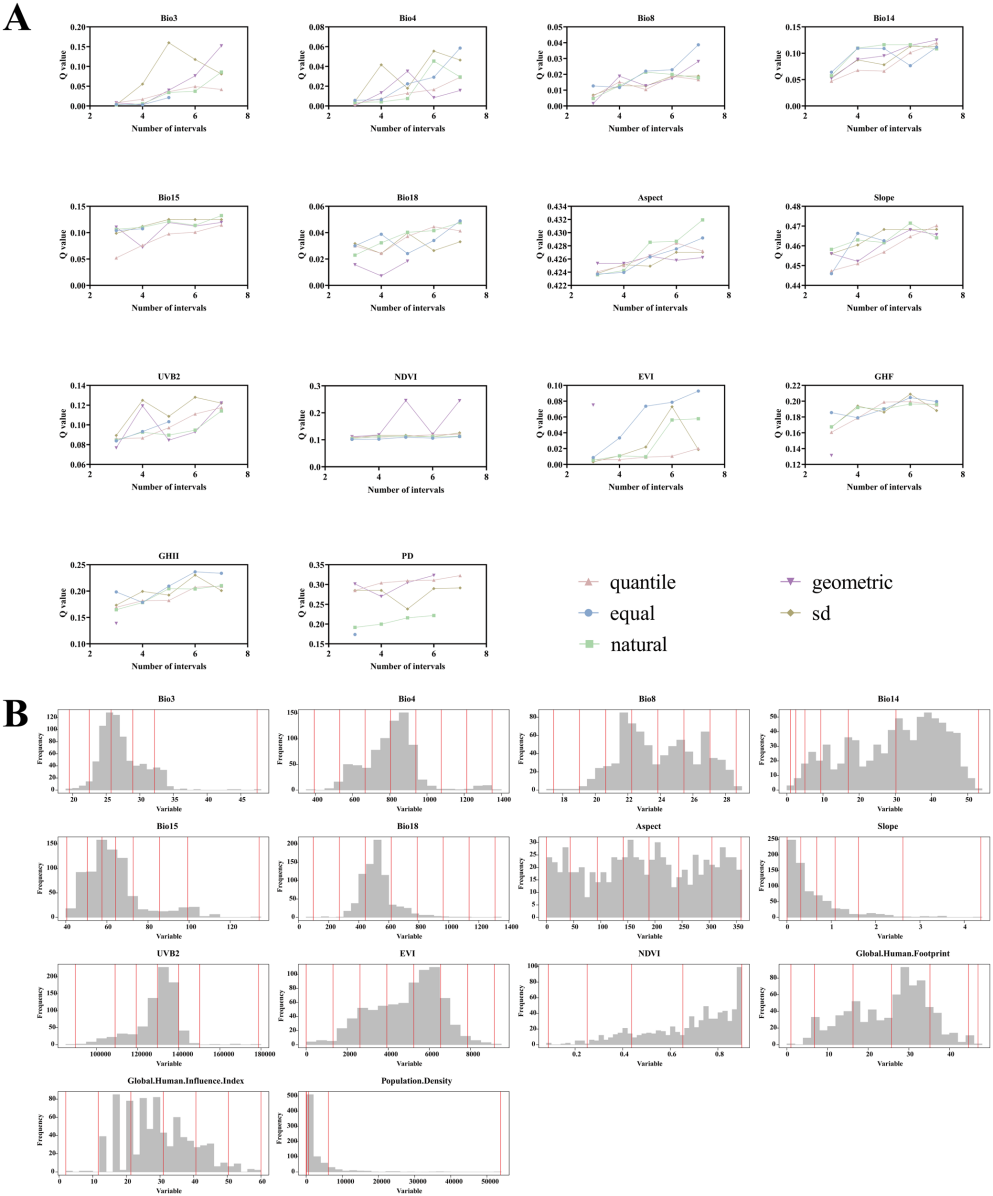
OPGD‐based exploration of explanatory variables for changes in habitat suitability in *B. xylophilus*. Note: (A) Variable discrete; (B) Partition results.

The results indicate that the optimal classification methods and the number of breakpoints for different environmental factors vary significantly, suggesting that environmental factors exhibit pronounced heterogeneity in their spatial distribution (Figure 6). Isothermality (Bio3) and NDVI were discretized using geometric and standard deviation (SD) methods across five intervals, respectively. Slope, GHII, and PD were discretized using equal, natural, and geometric methods across six intervals, respectively. UV‐B seasonality (UVB2) and GHF were discretized into six intervals using the SD method. Furthermore, the best classification method for temperature seasonality (Bio4), mean temp of wettest quarter (Bio8), precipitation of warmest quarter (Bio18), and enhanced vegetation index (EVI) was to use the equal method across seven intervals. Precipitation seasonality (Bio15) and Aspect were classified using the natural method in seven intervals, while precipitation of driest month (Bio14) was discretized using the geometric method in seven intervals. The findings suggest that different environmental factors have varying spatial expressions in their influence on *B. xylophilus* suitability.

#### 3.4.2 Detection of Influential Factors

**FIGURE 7:**
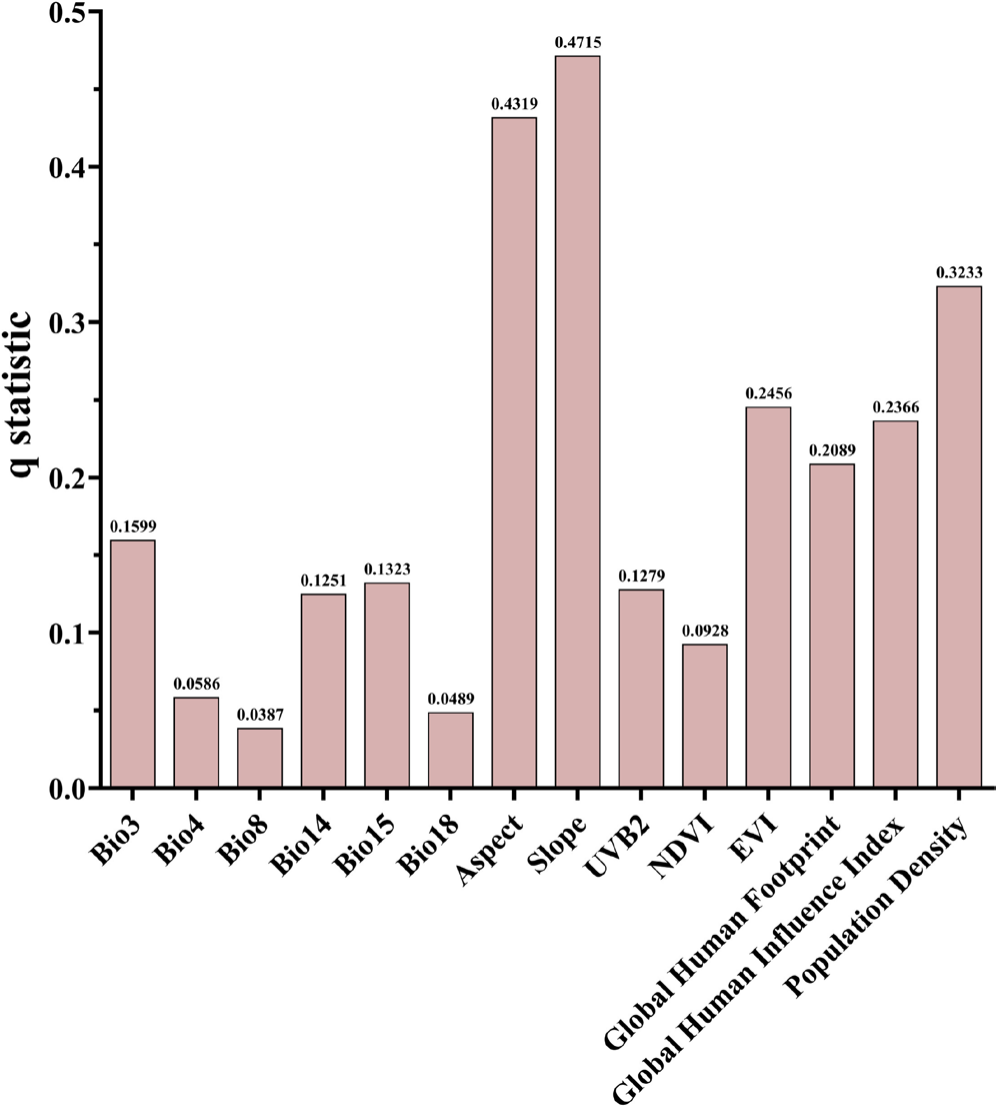
14 Explanatory power of environmental variables.

We applied the factor detector to determine the influence of each environmental variable on the habitat suitability of *B. xylophilus* (Figure 7). The variables were ranked by their explanatory power (*q*‐value) from highest to lowest: Slope > Aspect > PD > EVI > GHII > GHF > Bio3 > Bio15 > UVB2 > Bio14 > NDVI > Bio18 > Bio4 > Bio8. Among these, Slope and Aspect had the highest q‐values of 0.4715 and 0.4319, respectively, making them the two most influential environmental factors, with explanatory power exceeding 40%. This indicates that topographic features are the primary drivers of *B. xylophilus* habitat suitability. In contrast, Bio8 and Bio18 had relatively low q‐values, with explanatory power below 5%, suggesting their limited impact on the species' suitability. The results from the factor detector indicate that topographic features (Slope and Aspect), human activities (GHII and GHF), and vegetation indices (EVI and NDVI) are key factors influencing the habitat suitability of *B. xylophilus*. This highlights that the distribution of *B. xylophilus* is not only significantly influenced by natural topography but also regulated by human activities and vegetation conditions.

#### 3.4.3 Detection of Factor Interactions

**FIGURE 8:**
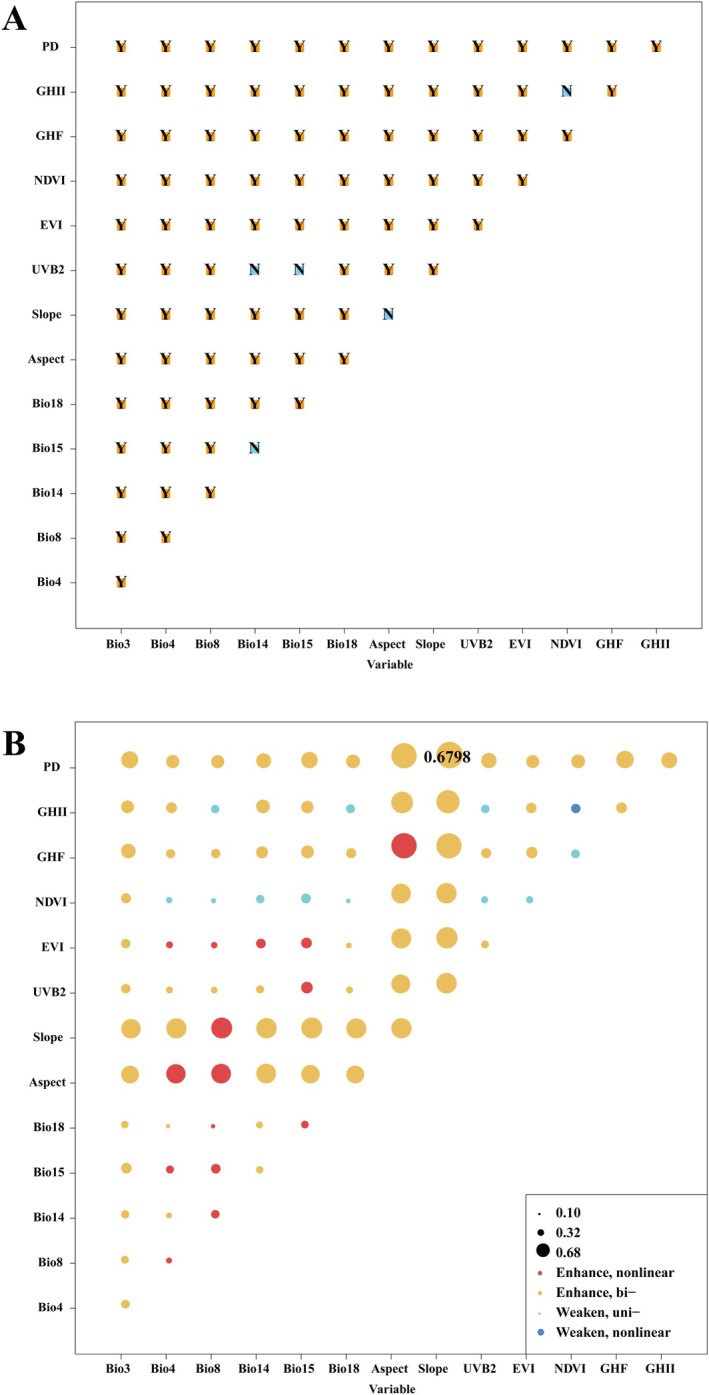
(A) The result of ecological detection; (B) Detecting the impact of drivers on vegetation change using OPGD model interactions. Note: *Y* represents a significant difference; *N* means no significant difference.

We used ecological detectors to evaluate whether there were statistically significant differences in the relative importance of 14 environmental factors influencing the distribution of *B. xylophilus* (Figure 8A). The results showed no significant differences between precipitation of the driest month (Bio14) with precipitation seasonality (Bio15) and UV‐B seasonality (UVB2), Aspect and Slope, and NDVI and GHII, indicating that these environmental factors have similar effects in explaining the distribution of *B. xylophilus*. However, significant differences were observed between all other pairs of environmental variables, including Bio3, Bio4, Bio8, Bio18, EVI, GHF, and PD. This highlights the significant influence of bioclimatic factors, vegetation indices, and human activities on the distribution of *B. xylophilus*. The ecological detector results suggest that these variables exhibit strong spatial differentiation effects, implying their central role in shaping habitat suitability patterns. This further supports their critical importance in ecological risk assessments.

In order to better understand the interactions between environmental factors, we assessed the association strength of interrelationships between environmental factors on habitat suitability of *B. xylophilus* using an interaction detector (Figure 8B). The results showed that the interactions between any two environmental factors primarily exhibited the following types: “Enhance, bi‐,” “Enhance, nonlinear,” “Weaken, uni‐,” and “Weaken, nonlinear.” Notably, no environmental factors acted completely independently in these interactions. Specifically, the “Enhance, bi‐” interaction between Slope and PD was the most significant, with a *q*‐value of 0.6798. This indicates that the combined influence of these two factors strongly explains the spatial heterogeneity of *B. xylophilus* distribution. It also suggests that slope and human activity (PD) work synergistically to enhance the explanatory power of habitat suitability. On the other hand, the interaction between NDVI and GHII was identified as “Weaken, nonlinear,” meaning that their combined effect weakened the explanatory power of habitat suitability compared to their individual effects, exhibiting a nonlinear relationship. This phenomenon might reflect the complexity or potential competitive relationship between NDVI and GHII in driving species distribution within the ecosystem, where they may inhibit each other in certain regions, leading to a combined effect lower than expected.

#### 3.4.4 Risk Detector

**FIGURE 9:**
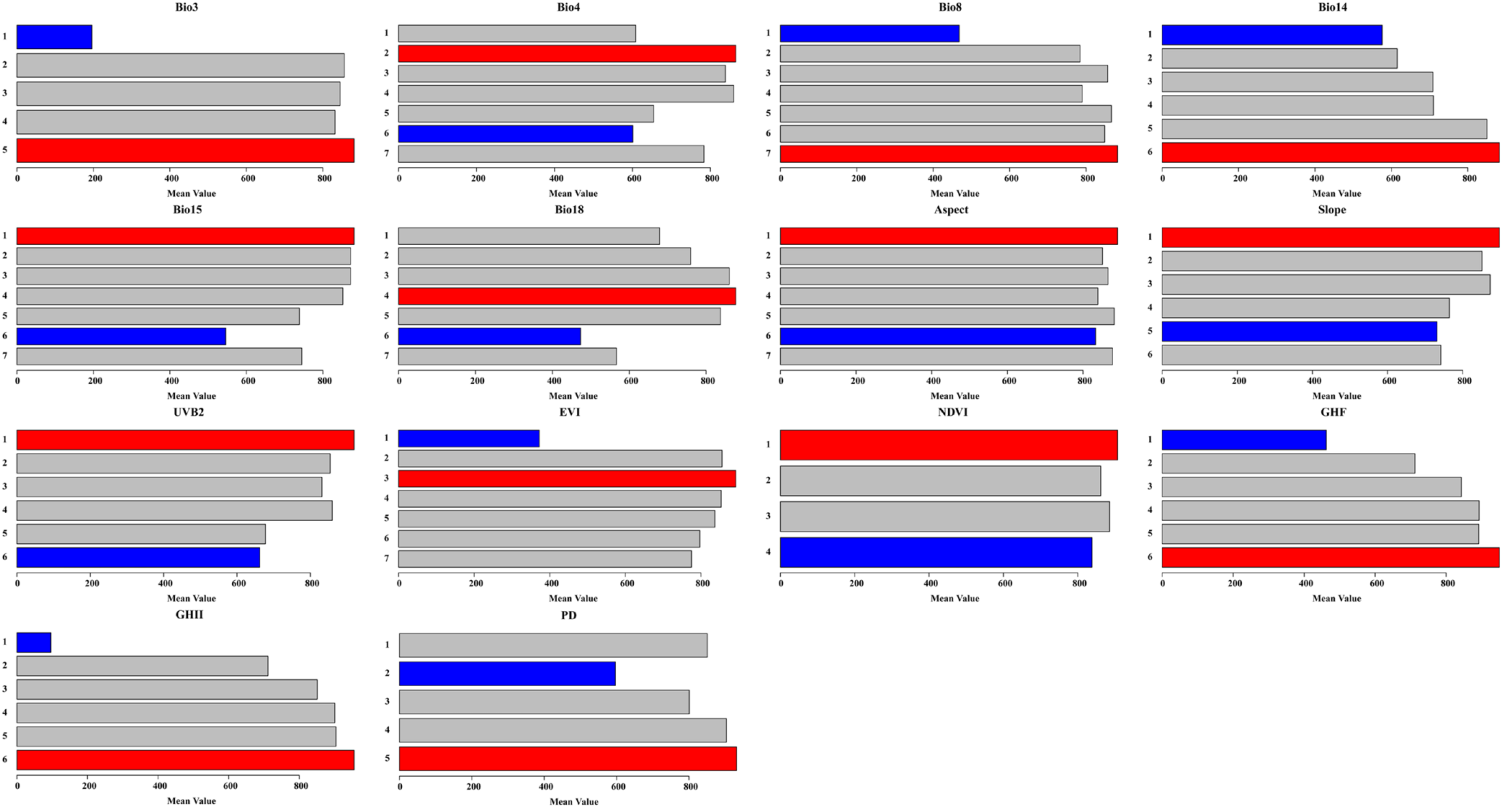
The risk mean values of OPGD‐based explanatory variables for exploration of habitat suitability of *B. xylophilus*. Note: Elevated values (red), intermediate values (gray), and decreased values (blue).

To explore the impact of regional attributes of driving factors on the suitability index of *B. xylophilus*, we calculated the risk contribution of these environmental factors across different ranges (Figure 9). Based on the results from the risk detector, we classified the habitat suitability of *B. xylophilus* into three levels: elevated values (red), intermediate values (gray), and decreased values (blue). The results showed significant differences in the influence of various driving factors on the suitability of *B. xylophilus* across different ranges of explanatory variables. Specifically, the suitability of *B. xylophilus* increased with the rise in bio3, bio8, bio14, GHF, GHII, and PD, and the increase of these driving factors contributed to the expansion of its suitable area. In contrast, the suitability of *B. xylophilus* decreased with the increase in UVB2 and NDVI, indicating that the increase in these factors might have an adverse effect on the suitable areas for the species. The increase in UVB2 and NDVI may lead to unfavorable habitat conditions, thus suppressing the distribution or suitability of the species.

## 4 Discussion

Habitat suitability for parasitic nematodes is strongly influenced by environmental factors, including bioclimatic variables, vegetation indices, topography, and human activities. These factors can affect nematode survival, dispersal potential, and host tree vulnerability. *B. xylophilus* is primarily dispersed by vector beetles and timber transport, which are strongly correlated with the intensity of human activities. Climatic variables such as temperature seasonality and precipitation patterns also affect developmental rates and overwintering rates (Pimentel et al. 2017; Li, Fan, et al. 2021). Zhang, Wang, et al. (2024a) used the Jackknife method in the MaxEnt model to identify Bio3, Bio4, Bio14, Bio15, Aspect, Slope, NDVI, GHII, and GHF as the primary factors influencing the potential distribution of *B. xylophilus*, with these factors contributing a cumulative rate of 86.4%. In this study, we similarly selected six bioclimatic factors (Bio3, Bio4, Bio8, Bio14, Bio15, and Bio18), three human activity factors (GHF, GHII, and PD), two topographic factors (Aspect and Slope), two vegetation factors (NDVI and EVI), and one solar radiation factor (UVB2). These factors work together to influence the distributional suitability of *B. xylophilus*, highlighting the complex role of multiple environmental factors in the prediction of its suitable habitat. The response curves further verified the effects of bioclimatic factors and human activities on the habitat suitability of *B. xylophilus*, especially with the increase in the intensity of human activities, suggesting that human activities may have carried *B. xylophilus* through production activities, which in turn contributed to its spread (CABI 2024; Zhang, Wang, et al. 2024a). Additionally, Ouyang et al. (2022) and Chen et al. (2024) used MAXENT and CLIMEX models to predict the main distribution areas of *B. xylophilus* in the current period, indicating that it is primarily distributed in South and North China. Meanwhile, the 2024 announcement from the National Forestry and Grassland Administration (NFGA) reported the discovery of *B. xylophilus* in Jilin and Gansu provinces (NFGA 2024). This study, using an ensemble model, further corroborates these findings, revealing that the potentially suitable areas for *B. xylophilus* are mainly concentrated in the northeast, southeast, and parts of the northwest regions in the current period. This result not only aligns with previous studies but also provides additional validation of the high predictability and accuracy of ensemble models in forecasting species distribution suitability.

Compared to traditional regression analysis methods, the geographical detector method offers a more detailed spatial differentiation and spatial analysis ability by comparing the distribution patterns of species suitability with environmental factors in space (Wang and Xu 2017). In this study, the optimized geographical detector model (OPGD) was used to discretize environmental variables influencing the distribution suitability of *B. xylophilus*, revealing the spatial heterogeneity of different environmental factors. The results indicate significant differences in the performance of various environmental factors under different discretization methods, emphasizing the complexity and diversity of environmental factors in spatial distribution. Furthermore, it suggests that the spatial distribution patterns of environmental factors vary across different regions, which may be closely related to factors such as geography, climate, and human activities. Factor detector analysis effectively quantifies the driving effects of various environmental factors on species distribution suitability (Liao et al. 2022). Through factor detector analysis, we found that Slope and Aspect are the most important influencing factors, with explanatory power exceeding 40%. This indicates that variations in terrain features have a significant impact on the suitability of *B. xylophilus*, likely through influencing moisture, soil conditions, and the thermal distribution of habitats, thereby affecting its living environment. Meanwhile, bioclimatic factors, vegetation indices, solar radiation, and human activities also play a significant role in regulating the suitability of *B. xylophilus*. Although their influence is relatively smaller compared to the terrain, they still exhibit significant spatial distribution patterns (Haynes et al. 2018; Zhang, Wang, et al. 2024b). This suggests that the distribution of *B. xylophilus* is not only controlled by natural terrain but also influenced by other environmental factors. The results from interaction detector analysis show that significant interactions exist between most environmental variables, highlighting the complex interrelationships among environmental factors. Notably, the interaction between environmental factors can either enhance or weaken the species' suitability. For instance, the interaction between Slope and PD significantly enhances the suitability of *B. xylophilus*, with a *q*‐value of 0.6798, indicating that the synergistic effect between the two factors plays a crucial role in influencing the distribution of *B. xylophilus*. This is likely due to the close relationship between changes in Slope and PD (such as urbanization and forestry activities), with their interaction potentially making habitat conditions more suitable for the distribution of *B. xylophilus* in certain regions (Zhang, Wang, et al. 2024a). In contrast, the interaction between NDVI and GHII exhibits a nonlinear weakening effect, which may be due to the combined effects of vegetation index and human activities, leading to a reduction in suitability. Specifically, areas with high human activity intensity may cause vegetation degradation or fragmentation, resulting in lower NDVI values. This degradation in vegetation cover can reduce habitat availability and quality, thereby negatively affecting the survival and reproduction of *B. xylophilus*. It is also important to consider that forests already affected by pine wilt may show lower NDVI values due to tree mortality. Moreover, anthropogenic activities such as urban expansion, deforestation, and agricultural development can significantly modify local microclimatic conditions and intensify ecological disturbances. These changes can undermine the ecological stability of regions that inherently possess high environmental suitability, thereby diminishing their capacity to support sustainable populations of *B. xylophilus*. This finding suggests that in some areas, human activities might have a negative effect on the vegetation index, limiting the suitability for *B. xylophilus*. Meanwhile, significant nonlinear weakening interaction effects between bioclimatic factors and human activities were found, suggesting that human activities might exacerbate climate change to some extent, further reducing the distribution suitability of *B. xylophilus* (Xiao et al. 2024). Therefore, considering the interactions between environmental factors will help to more accurately predict species distribution. Additionally, in the risk detector analysis, it was found that the risk contribution of different environmental factors varies significantly across different intervals. Specifically, increases in Bio3, GHF, GHII, and PD promote an increase in the suitability of *B. xylophilus*, while factors such as UVB2 and NDVI exhibit inhibitory effects, leading to a decrease in suitability. This suggests that an increase in UVB2 may bring more ultraviolet radiation, thereby altering the ecosystem's microclimate and limiting the survival of *B. xylophilus* (Zhang, Wang, et al. 2024a). A larger NDVI value likely indicates more abundant vegetation, which may change the habitat conditions of *B. xylophilus*, further reducing its suitability. The results of the study not only provide important spatial data for understanding the ecological suitability of *B. xylophilus*, but also provide methodological references for other ecological studies and species distribution modeling, and further emphasize the complex role of environmental factors in predicting the suitability of species, which will help to formulate a more precise ecological protection and species prevention and control strategy.

In this study, we combined two methods, ensemble model and geographical detector, to assess the influence of 14 environmental factors on the potential suitable distribution of *B. xylophilus*. The two methods portrayed the influence of the environmental factors from different dimensions, and the results showed that the importance of the environmental variables generated by the different methods differed somewhat, reflecting the complementarity of methodologies and the complexity of ecological processes. The ensemble model relies on occurrence point data and emphasizes the predictive power of variables at the point scale for modeling species suitability by fitting nonlinear relationships between species distribution probabilities and environmental factors. Its results show that anthropogenic‐related factors (e.g., PD, GHII, and GHF) have high contribution values, while some climatic variables (e.g., Bio3, Bio4, Aspect, etc.) have low weights. This suggests that human activities have played a key role in the dispersal and colonization of *B. xylophilus* in the current period, and may have affected the suitability of *B. xylophilus* by disturbing ecosystem structure and expanding dispersal pathways (e.g., timber transport and infrastructure construction). In contrast, the geographic detector placed more emphasis on the explanatory power of environmental factors on spatial patterns of species suitability, reflecting the dominant role of variables in spatial differentiation. The results of the analyses showed that the variables of slope, aspect orientation, vegetation index (EVI), and PD had high q‐values, indicating that these factors had significant spatial driving effects on the potential distribution of *B. xylophilus* at large regional scales. Particularly, although slope and aspect orientation had low numerical contributions in the ensemble model, their high explanatory power in the geographical detector suggested that topographic factors might indirectly regulate the spatial dispersal of *B. xylophilus* by influencing the distribution pattern of host pine trees or the activity path of vector insects. It is noteworthy that PD was identified as a significant variable in both approaches, showing a highly consistent effect of PD on *B. xylophilus* dispersal across scales and modeling mechanisms, a result that also coincides with recent field monitoring of frequent *B. xylophilus* invasions in areas of high human activity. The consistency of such variables not only enhances the robustness of the results, but also provides data support for the inclusion of human disturbance in ecological risk early warning systems. Thus, combining the predictive power of ensemble model with the spatial resolution of geographical detector helps to comprehensively identify the key environmental drivers of *B. xylophilus* invasion at both micromechanism and macropattern levels. The integrated framework provides a theoretical reference for the in‐depth understanding of species–environment relationship, and an effective tool for improving the accuracy of spatial prediction of exotic pests and the regional deployment of scientific prevention and control strategies. In the future, we can further explore the integration of these two approaches to construct more interpretable multiscale species distribution models, and promote the in‐depth translation of ecological modeling results into management practices.

This study reveals the mechanisms by which multiple environmental factors influence the potentially suitable distribution of *B. xylophilus* and identifies key environmental variables that drive its spatial distribution pattern. These ecological findings not only deepen the understanding of the ecological niche characteristics of *B. xylophilus* but also provide a theoretical basis for the development of scientific and efficient forest pest management policies. Based on the prediction results, high‐suitability areas can be designated as key regulatory areas, the monitoring network can be laid out on a priority basis, the quarantine supervision of timber transport and circulation can be strengthened, and the monitoring of forest health can be carried out in combination with remote sensing technology in order to enhance the ability of early detection and intervention of epidemics. Meanwhile, key environmental variables identified should be incorporated into the forestry ecological monitoring index system, and a dynamic risk assessment and early warning model should be constructed to provide precise early warning for potential future outbreak areas. For high‐risk areas, the optimization and adjustment of forest stand structure can be implemented, and three‐dimensional and intelligent integrated management measures can be promoted by combining drone inspections, manual ground inspections, and biological control. In addition, this study can also provide data support for improving the forest quarantine system, designating high‐suitability areas as key quarantine supervision objects, strengthening the risk control of foreign timber transfer, and preventing the transregional spread of *B. xylophilus*. In conclusion, embedding the suitability prediction model constructed in this study into the prevention, control, and management system of *B. xylophilus* will help to improve China's scientific decision‐making level and intelligent management ability in pest prevention, control, and emergency response.

Some limitations and uncertainties remain in this study. Firstly, the environmental variables selected for this study may not cover all the factors that may affect the distribution of *B. xylophilus*. For instance, soil type, air humidity, wind speed, and other variables may also have a significant impact on ecological suitability, but these factors were not considered in this study. Therefore, the model may not fully capture the complex interactions in the ecosystem, which could lead to less precise species distribution predictions under certain regional or environmental conditions. Future studies could further incorporate additional environmental factors to enhance the model's predictive capability. Moreover, this study fails to adequately consider the dynamic effects of climate change on species distribution. As global climate change intensifies, factors such as rising temperatures and changing precipitation patterns will directly or indirectly influence the suitable habitats of *B. xylophilus*. Therefore, future research should take into account future climate scenarios to more comprehensively assess the impact of climate change on invasive species, thus providing more complete theoretical support for species control. Additionally, although it covers a broad geographical range, significant spatial heterogeneity may still exist between geographic regions, especially for species distribution prediction in localized areas. Future studies could combine higher‐resolution spatial data with detailed observations from meteorological stations to better reveal regional differences, thereby improving the local predictive power of the models and providing more accurate strategies for species control and ecological management.

## 5 Conclusions

This study comprehensively assessed the distribution suitability of *B. xylophilus* and its main environmental driving factors using ensemble modeling techniques and the optimized parameter geographical detector. The research shows that a variety of environmental factors, such as bioclimate, vegetation, topography, solar radiation, and human activities, have a significant impact on its suitable habitat, and the areas with high suitability are mainly concentrated in South China, North China, and a portion of Northeast China. Furthermore, geographical detector analysis revealed the spatial heterogeneity of environmental factors and their interactions with *B. xylophilus* habitat suitability, indicating that these factors influence its distribution by either enhancing or suppressing suitability. Notably, the interaction between human activity and natural environmental factors plays a complex regulatory role in the distribution of *B. xylophilus*. Additionally, in‐depth analysis using other components within the geographical detector framework (e.g., risk, factor, and ecological detection) identified the specific mechanisms by which these environmental factors affect *B. xylophilus* habitats, revealing differences and spatial patterns in their effects across regions, providing valuable insights into the regional variation of species distribution. The results of this study not only provide an important theoretical foundation for research on the ecological suitability of *B. xylophilus* but also offer scientific guidance for regional ecological protection and species control strategies.

## Author Contributions

**Liang Zhang:** conceptualization (equal), formal analysis (equal), methodology (equal), software (equal), validation (equal), visualization (equal), writing – original draft (equal), writing – review and editing (equal). **Jie Li:** data curation (equal), methodology (equal). **Chaokun Yang:** data curation (equal), validation (equal). **Ping Wang:** data curation (equal), funding acquisition (equal), investigation (equal). **Guanglin Xie:** data curation (equal), investigation (equal), visualization (equal). **Wenkai Wang:** funding acquisition (equal), project administration (equal), resources (equal), supervision (equal).

## Conflicts of Interest

The authors declare no conflicts of interest.

## Supporting information


Data S1.

Data S2.

## Data Availability

I confirm that the Data Availability Statement is included in the main file of my submission, and that access to all necessary data files is provided to editors and reviewers.
